# A transient wave of BMP signaling in the retina is necessary for Müller glial differentiation

**DOI:** 10.1242/dev.118745

**Published:** 2015-02-01

**Authors:** Yumi Ueki, Matthew S. Wilken, Kristen E. Cox, Laura B. Chipman, Olivia Bermingham-McDonogh, Thomas A. Reh

**Affiliations:** 1 Department of Biological Structure, University of Washington, Seattle, WA 98195, USA; 2 Molecular and Cellular Biology Program, University of Washington, Seattle, WA 98195, USA

**Keywords:** Neurogenesis, Glia, Smad, Id1, Mouse

## Abstract

The primary glial cells in the retina, the Müller glia, differentiate from retinal progenitors in the first postnatal week. CNTF/LIF/STAT3 signaling has been shown to promote their differentiation; however, another key glial differentiation signal, BMP, has not been examined during this period of Müller glial differentiation. In the course of our analysis of the BMP signaling pathway, we observed a transient wave of Smad1/5/8 signaling in the inner nuclear layer at the end of the first postnatal week, from postnatal day (P) 5 to P9, after the end of neurogenesis. To determine the function of this transient wave, we blocked BMP signaling during this period *in vitro* or *in vivo*, using either a BMP receptor antagonist or noggin (Nog). Either treatment leads to a reduction in expression of the Müller glia-specific genes *Rlbp1* and *Glul*, and the failure of many of the Müller glia to repress the bipolar/photoreceptor gene *Otx2*. These changes in normal Müller glial differentiation result in permanent disruption of the retina, including defects in the outer limiting membrane, rosette formation and a reduction in functional acuity. Our results thus show that Müller glia require a transient BMP signal at the end of neurogenesis to fully repress the neural gene expression program and to promote glial gene expression.

## Abstract (precis)

**Summary**: BMP signalling is transiently activated in the postnatal mouse retina to terminate the neurogenic program and promote the expression of glial-specific genes.

## INTRODUCTION

Several signaling pathways regulate the production and differentiation of glial cells in the central nervous system of vertebrates. For the astrocyte lineage, the best characterized of these signaling pathways is CNTF/LIF, acting through the gp130 receptor and the JAK-STAT intracellular effectors. Addition of CNTF/LIF to dissociated cell cultures of embryonic brain causes an increase in the number of astrocytes that differentiate *in vitro* (Bonni et al., 1997; Nakashima et al., 1999a,b). Astrocyte differentiation is initiated during brain development by an increase in gp130 signaling in radial glia of embryonic brain (He et al., 2005), leading to STAT3 activation of *Gfap* and other astrocyte genes; this developmental expression of gp130 in the radial glia requires MEK signaling (Li et al., 2012). BMP signaling is also important for astrocyte development. Addition of BMP to dissociated cultures of embryonic brain promotes astrocyte numbers and gene expression (Mabie et al., 1997; Grinspan et al., 2000; See et al., 2004). *In vivo* overexpression of BMP4 in developing mouse brain leads to an increase in astrocyte number (Gomes et al., 2003); mice deficient in both Bmpr1a and Bmpr1b show a 25-40% decrease in the number of astrocytes expressing either S100β or GFAP (See et al., 2007).

In the retina, a specialized type of glia, the Müller glia, carries out some of the same functions as astrocytes in other regions of the central nervous system (Bringmann et al., 2006). Müller glia are generated at the end of neurogenesis by multipotent retinal progenitors (Turner and Cepko, 1987; Livesey and Cepko, 2001) and are related to astrocytes in their gene expression (Nelson et al., 2011). Several studies have reported that, like in astrocytes, CNTF/LIF signaling promotes Müller glial differentiation during retinal development (Goureau et al., 2004; Rhee and Yang, 2010); however, the role of BMP signaling in Müller glial differentiation is less clear. Blocking or activating BMP signaling in chick (Huillard et al., 2005) or mouse embryos (Kuribayashi et al., 2014) leads to a loss or increase in the number of Müller glia, respectively. However, these studies concluded that the effects of BMP were on the retinal progenitors rather than on Müller glia. We have therefore analyzed the time course of BMP signaling during a period of retinal development in which Müller glial differentiation occurs in mice, the first postnatal week. We find a wave of phospho-Smad 1/5/8 expression in the inner nuclear layer (INL) of the retina from postnatal day (P) 5 to P9. This transient increase in Smad1/5/8 phosphorylation is probably mediated through Bmpr1a, as this receptor is highly expressed in Müller glia at this stage. Inhibition of BMP signaling during this period disrupts Müller glial development resulting in permanent disorganization of the retina. Overall, our data show that Müller glia require a transient BMP signal to consolidate their differentiation.

## RESULTS

### Smad1/5/8 phosphorylation increases in the INL between P6 and P8

**Fig. 1.**
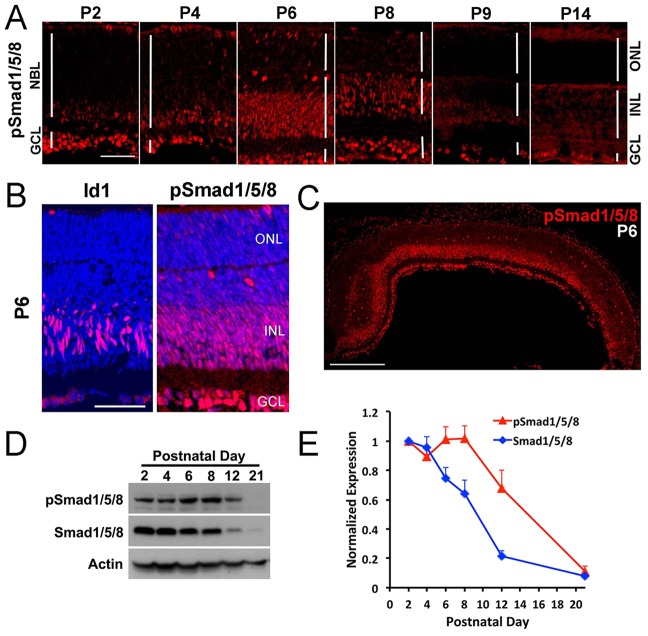
**BMP signaling is activated transiently in the INL between P5 and P8.** (A) pSmad1/5/8 immunostaining at indicated postnatal ages. NBL, neuroblastic layer; GCL, ganglion cell layer; INL, inner nuclear layer; ONL, outer nuclear layer. Scale bar: 50 µm. (B) Higher magnification view comparing the expression of Id1 (a Müller glial marker) with pSmad1/5/8 in a similar section Scale bar: 50 µm. (C) Activation of Smad1/5/8 (pSmad1/5/8) in the INL of one half of the retina. Scale bar: 250 µm. (D) Western blot of retinas harvested at indicated ages with actin loading control. (E) Western blot quantification. pSmad1/5/8 (red) or Smad1/5/8 (blue) normalized to actin. Error bars are s.e.m. (*n*=3).

To determine whether BMP signaling participates in the differentiation of Müller glia, we monitored the phosphorylation of Smad1/5/8 by immunofluorescence and western blots in the first two postnatal weeks. We verified the specificity of anti-pSmad1/5/8 antibody using a BMP inhibitor (supplementary material Fig. S1). At P2 the ganglion cells were labeled (Fig. 1A; GCL), but little or no pSmad1/5/8 was detected in other retinal cells (Fig. 1A; NBL). At P4 there is an increase in pSmad1/5/8 labeling in the inner part of the NBL, probably amacrine cells. At P6 and P8, there is a large increase in pSmad1/5/8 labeling of many cells in the INL. Many of these cells are Id1+ Müller glia (Fig. 1B), although other cells in the INL, probably amacrine cells, are also labeled. This increase in pSmad1/5/8 labeling in the INL is transient. However, by P9, only low levels of pSmad1/5/8 labeling are observed. The wave-like spread of this signal can be better appreciated in a lower magnification image taken at P6 (Fig. 1C), which shows that the pSmad1/5/8 signal extends across the retina at this age. Throughout this period, there is also pSmad1/5/8 immunofluorescence in the ganglion cells that persists until at least P14 (Fig. 1A; GCL). Western blot analysis of total Smad1/5/8 and pSmad1/5/8 confirmed the immunofluorescence analysis. Total Smad1/5/8 is highest at P2 and declines during this period, becoming undetectable by P21, whereas the level of pSmad1/5/8 reaches its maximum at P6 and P8 (Fig. 1D,E).

**Fig. 2.**
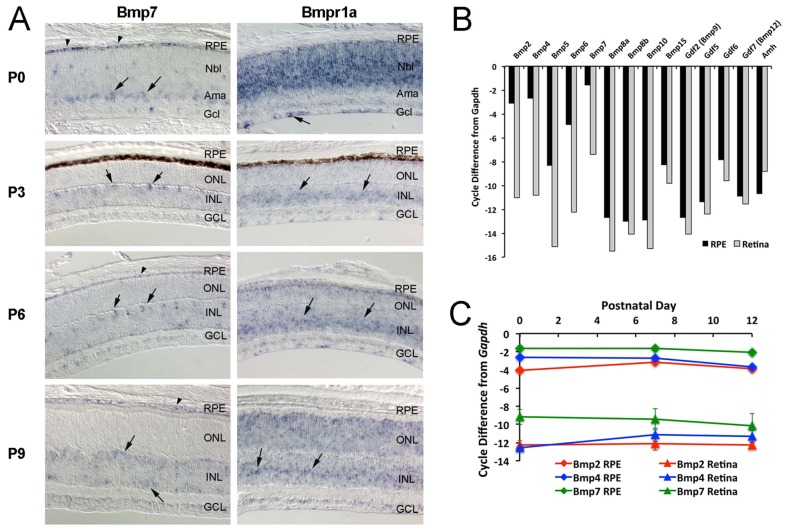
**BMP receptor and ligand expression in the developing retina.** (A) (Left) Representative images of *in situ* hybridization for *Bmp7*. *Bmp7* mRNA was detected in the RPE (arrowhead), amacrine and horizontal cells (arrows). (Right) Representative images of *in situ* hybridization for *Bmpr1a* at the ages shown. *Bmpr1a* mRNA was detected in retinal progenitors at P0 (and in some ganglion cells, arrows at P0) and in the INL, where Müller glial nuclei were located from P3-P9 (arrows). (B) Ligands of BMP receptors at P7 assessed by qRT-PCR. Bars represent the value from a pooled sample normalized to *Gapdh*. (C) *Bmp2*, *4* and *7* were measured at P0, P7 and P12. Error bars are s.e.m. (*n*=3 for retinas). RPE samples were pooled from 12-18 eyes at each age.

To determine which BMP receptor and ligand(s) mediate this transient increase in pSmad1/5/8, we carried out *in situ* hybridization for *Bmpr1a* and *Bmpr1b*. Fig. 2A shows expression of *Bmpr1a* in the INL at P0, and in the developing Müller glia at P3 and P6. At P9, *Bmpr1a* is expressed throughout the retina, including the developing Müller glia. *Bmpr1b* is also expressed in the retina at P6 (supplementary material Fig. S2A); however, it is expressed only in the ventral part of the retina (Liu et al., 2003) and thus is unlikely to be mediating the transient Smad1/5/8 phosphorylation that occurs throughout the retina at P6. To identify the possible ligands driving the transient increase in pSmad1/5/8 during this period of retinal development, we carried out quantitative RT-PCR (qRT-PCR) for all known ligands able to activate the BMP receptors (Mueller and Nickel, 2012). We tested both retina and the retinal pigment epithelium (RPE), as previous studies have shown the RPE to be a source of several members of the Bmp family (Belecky-Adams and Adler, 2001; Trousse et al., 2001) (Fig. 2B). Our results show that *Bmp2*, *4*, *6* and *7* are strongly expressed in the RPE, whereas *Bmp7* was most highly expressed in the retina, although its level in the retina by qRT-PCR was many fold lower than its expression in the RPE (Fig. 2B). *In situ* hybridization for *Bmp4* and *Bmp7* confirmed expression of both in the RPE and the ciliary epithelium (supplementary material Fig. S2B,C). In addition, *Bmp7* was expressed in amacrine cells at P0, horizontal cells at P3 and P6, and possibly in bipolar cells at P9 (Fig. 2A). Although the *in situ* results and qRT-PCR are qualitatively consistent, the RPE expresses much higher levels of these ligands, and this is not apparent in the less quantitative *in situ* patterns. Overall, our results suggest that the ligand responsible for activation of this pathway in the retina is most likely Bmp7, either secreted by the RPE to activate the apical surfaces of the Müller glia, or locally within the retina via other inner retinal neurons.

### BMP signaling is necessary for Müller glial differentiation

**Fig. 3.**
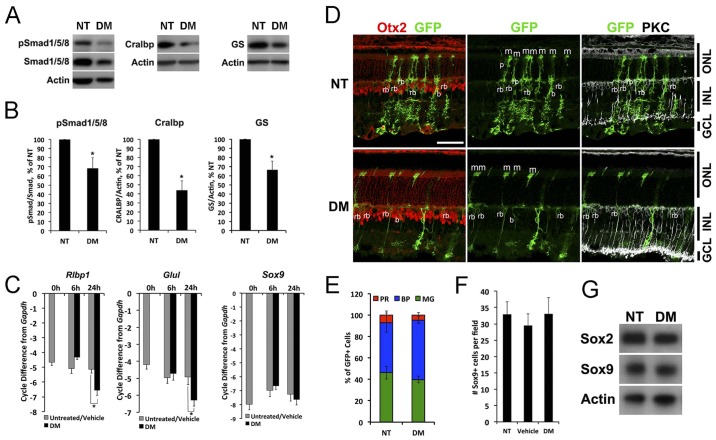
**Inhibition of BMP signaling at P6 destabilizes Müller glial phenotype.** (A) BMP inhibitor dorsomorphin (DM) was injected intravitreally at P6 and collected after 24 h. The other eye was uninjected (NT). (B) Western blot quantification. Error bars are s.e.m. *P<0.05 with paired *t*-test (*n*=5). (C) P6 retinal explants were treated with vehicle or DM for 0, 6 and 24 h and collected for qRT-PCR. Error bars are s.e.m. (*n*=4). *P<0.05 with *t*-test. (D) *Ascl1* lineage tracing of cells at P4, treated with DM at P6, collected at P21. Otx2 (bipolar cell, b; photoreceptor marker, p, red) and PKC (rod bipolar cell marker, rb, white). m, Müller glia. Scale bar: 50 µm. (E) Quantification of GFP+ Müller glia (MG), bipolar cells (BP) and photoreceptors (PR) in control and DM-treated Ascl1 lineages. Error bars are s.e.m. (*n*=5). (F) The number of Sox9+ nuclei in the INL. Error bars are s.e.m. (*n*≥7 per group). (G) BMP inhibitor DM was injected intravitreally at P6 and collected after 24 h; western blots were performed for Sox9 and Sox2.

We next tested whether inhibition of BMP signaling at the end of the first postnatal week affects Müller glial differentiation. We injected the BMP receptor antagonist dorsomorphin (DM) intravitreally in P6 mice and analyzed retinas 24 h later. The intravitreal injection of DM was effective at reducing pSmad1/5/8 levels (Fig. 3A). We found that the Müller glial-specific proteins Cralbp (Rlbp1 – Mouse Genome Informatics) and glutamine synthetase (GS) were significantly reduced in the DM-treated retinas after 24 h (Fig. 3A,B). A representative western blot is shown in Fig. 3A and quantification of three blots is shown in Fig. 3B. We also tested explants for both cellular retinaldehyde-binding protein (*Rlbp1*) and GS (*Glul*) gene expression by qRT-PCR after 6 and 24 h *in vitro*. In the DM-treated explants, both of these Müller glial genes are significantly reduced by 24 h (Fig. 3C). Although more difficult to quantify, we also found an apparent reduction in immunofluorescence when sections of treated retinas were labeled with antibodies against either GS or Cralbp (supplementary material Fig. S3).

Although glial differentiation markers were reduced by inhibition of BMP signaling, a transcription factor specifically expressed in Müller glia, Sox9, was unchanged by qRT-PCR or western blot (Fig. 3C,G). To confirm this result, we carried out intravitreal injections of DM at P6 and sacrificed the mice after a two-day or two-week survival period. Sections of the retinas were labeled for Sox9 and the Müller glia were quantified. When analyzed at P8, there was no significant difference in the number of Sox9+ cells in the DM-treated retinas (DM=62.4±10.6; vehicle=41.0±2.6; NT=41.75±4.7 per field). At P21, we also found no significant difference in the number of Sox9+ cells in retinas from eyes injected with DM when compared with either the vehicle-treated or untreated retinas (NT=32.9±3.9, vehicle=29.6±3.6, DM=33.1±5.0 per field). These results suggest that the transient BMP signal in the inner retina from P6 to P8 is important for glial gene expression, but not for the determination of Müller glial fate.

To further investigate the effects of BMP inhibition in the postnatal retina, we traced the lineage of the late-generated cohort of retinal cells with *Ascl1^CreERT2^* knock-in mice (Kim et al., 2011) crossed with Cre-dependent mTmG reporter mice (Muzumdar et al., 2007). *Ascl1^CreERT2^* is expressed in the late progenitors of the retina, and previous lineage-tracing studies have shown that, at P0, these cells will give rise to the ‘late-generated’ cohort of retinal neurons [bipolar cells, amacrine cells, rod photoreceptors and Müller glia (Brzezinski et al., 2011)]. For our experiments, Ascl1+ cells were traced with a single injection of tamoxifen at P4. Intravitreal injection of DM was then performed at P6 to inhibit the transient activation of BMP signaling, and the eyes were collected at P21 for immunohistochemistry. Cells were identified by morphology and immunofluorescence for cell type-specific markers. We counted the number of GFP+ Müller glia, bipolar cells and photoreceptors from randomly selected images taken from the central retina (Fig. 3D). We found that, at P4, the Ascl1+ progenitor lineage is composed of rod photoreceptors, bipolar cells and Müller glia (Fig. 3E). The proportions of Müller glia, bipolar cells and photoreceptors were not significantly different between DM-treated and untreated (NT) retinas. These results further support the conclusion that the inhibition of transient BMP signaling at P6 does not affect the cell fate determination in the late-staged progenitors.

**Fig. 4.**
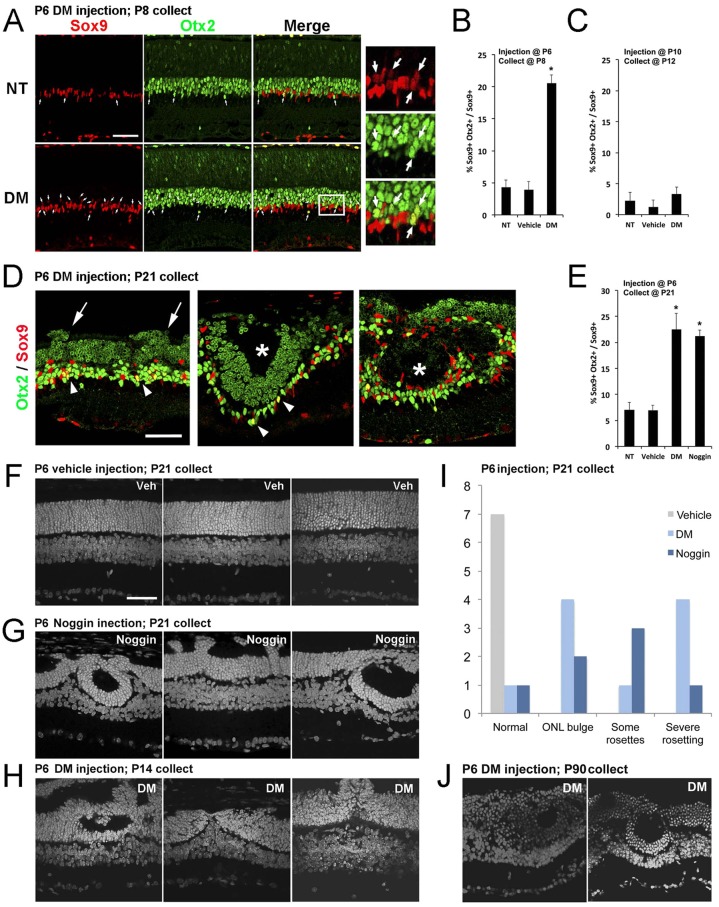
**Inhibition of BMP signaling at P6 leads to long-term changes in Müller glia and retinal structure.** (A) DM injected at P6, collected at P8 and labeled for Otx2 (green) and Sox9 (red). Arrows: Otx2+/Sox9+ cells. Box shows image at higher magnification. Scale bar: 50 µm. (B) Otx2+/Sox9+ cells/total Sox9+ cells, with DM injected at P6 and retinas collected at P8. (C) Otx2+/Sox9+ cells/total Sox9+ cells, with DM injected at P10 and retinas collected at P12. *P<0.05 with *t*-test (*n*=6-17 per group). (D) DM injected at P6, collected at P21. DM-treated retinas contained ‘mushroom-like’ bulges of photoreceptors (arrows) or rosettes (asterisks), and Otx2+/Sox9+ cells (arrowheads). (E) Otx2+/Sox9+ cells/total Sox9+ cells, with DM or Nog injected at P6 and retinas collected at P21. (F) Vehicle injection at P6 does not cause this phenotype. (G,H,J) Examples of retinas injected at P6 with DM or Nog and collected at P14 (H), P21 (G) or P90 (J). (I) Graph of morphological changes from P6 DM or Nog injections, P21 collection.

Although the overall number of Müller glia was not changed by the P6 DM treatment, there were several striking changes in these cells. Double-labeling the retinas for Sox9 and for Otx2, a transcription factor normally present only in photoreceptors and bipolar cells (Nishida et al., 2003), revealed a distinct phenotype in the DM-treated retina (Fig. 4A,B). In the normal retina, Otx2 and Sox9 are rarely co-expressed in the same cell (Fig. 4A; NT arrows). Whereas nearly all Müller glia expressed Sox9, only ∼5% of the Müller glia expressed the neural transcription factor Otx2 (Fig. 4B,C). However, in the retinas treated with DM, ∼20% co-expressed both markers. This effect was present as early as 2 days after the DM treatment (Fig. 4A,B), and persisted in animals analyzed weeks later at P21 (Fig. 4E). The effects on Müller glia only occurred if the DM injection was given at the time of the Smad1/5/8 activation. When the injection was made at P10, Müller glia did not increase their Otx2 expression (Fig. 4C). We also tested whether another inhibitor of BMP signaling, noggin (Nog), would have the same effect, and found that injections of Nog at P6 lead to a similar number of Otx2/Sox9 double-positive cells (Fig. 4E). Moreover, an additional anti-Otx2 antibody gave the same results (supplementary material Fig. S4). These results suggest that Müller glia require the transient BMP signaling at the end of neurogenesis to stabilize their phenotype and repress the neural gene *Otx2*.

**Fig. 5.**
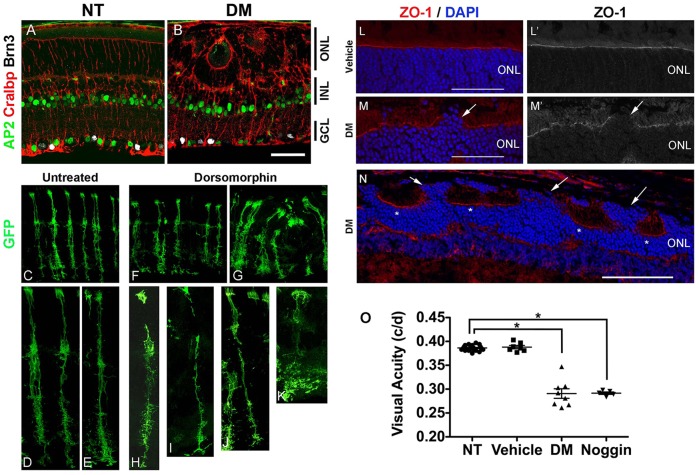
**Inhibition of transient BMP-Smad1/5/8 signaling during postnatal development causes permanent disruption in retinal morphology and function.** (A,B) AP2+ amacrine cells (green) and Brn3+ ganglion cells (white) in DM-treated P6 retinas (B) compared with NT (A). Müller glial marker Cralbp (red). Scale bar: 50 µm. (C-K) Examples of Müller glial morphology from *Ascl1* lineage retinas treated at P6 with DM or vehicle and collected at P21. (L-N) ZO-1 (red) labels OLM in untreated (L,L′) and DM-treated (M,M′,N) retinas. Arrows, gaps in OLM; asterisks, regions of intact OLM. Scale bars: 50 µm in L,M; 100 µm in N. (O) Vehicle, DM or Nog was injected at P6 and the visual acuity of each eye was measured at P28. Error bars are s.e.m. *P<0.05, *t*-test (*n*>5 per group).

Even more striking were changes in the overall retinal structure in the treated retinas. In approximately one-third of the treated retinas, the outer nuclear layer (ONL) contained ‘mushroom-like’ bulges of photoreceptors (Fig. 4D,I). In the majority of the treated retinas there were rosettes, and in another third, severe morphological disruptions occurred across the entire retina (Fig. 4D,G-I; supplementary material Fig. S5). These changes were observed in either DM- or Nog-treated retinas collected as early as P14, but became more pronounced by P21 and persisted for at least 90 days (Fig. 4I,J; Table 1). Although the retinal structure was abnormal, labeling for inner retinal neurons did not reveal any apparent differences (AP2+ amacrine cells, Fig. 5A,B; AP2); counts of ganglion cells showed no difference between treated and control retinas (supplementary material Fig. S6B). There were also no apparent differences in retinal vasculature (supplementary material Fig. S6A; lectin) or in the associated astrocytes (supplementary material Fig. S6A; GFAP).

**Table 1.**
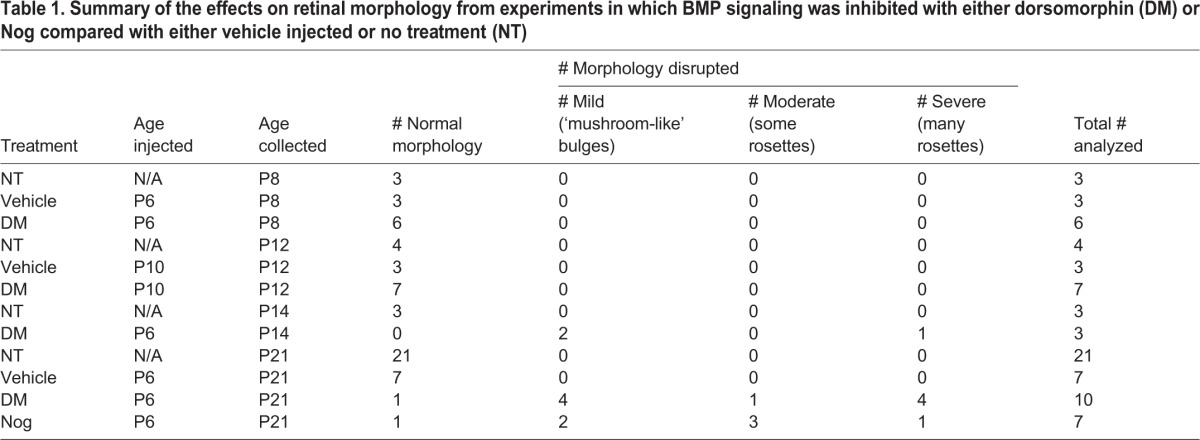
**Summary of the effects on retinal morphology from experiments in which BMP signaling was inhibited with either dorsomorphin (DM) or Nog compared with either vehicle injected or no treatment (NT)**

The formation of bulges in the ONL and rosettes is characteristic of many mutations in outer limiting membrane (OLM) genes. Therefore we analyzed whether there were defects in the OLM with an antibody against ZO-1 (Tjp1 – Mouse Genome Informatics). The DM-treated retinas had clear gaps in the ZO-1 labeling in the regions of rosette formation (Fig. 5M,N). We observed these gaps in both rosetted regions and in the positions of the ‘mushroom-like’ bulges, suggesting that some aspects of the phenotype we observe with BMP inhibition at P6 are due to a reduction in glial expression of OLM proteins. We also examined Müller glial morphology in DM-treated retinas, using the same *Ascl1^CreERT2^* knock-in mice used for the lineage studies. In the untreated retina, the typical Müller glial morphology was observed, but in the DM-treated retina, the morphology was more variable: some cells were long and spindly with few processes, whereas others were short with very abnormal branching patterns (Fig. 5C-K).

The inhibition of BMP signaling with a single injection of either DM or Nog at P6 leads to a reduction in Müller glial gene expression, a failure to suppress the photoreceptor/bipolar transcription factor Otx2 and a striking disruption of retinal structure. We next tested whether the inhibition of BMP signaling in the postnatal retina has an effect on retinal function (Fig. 5O). Visual acuity of each eye was measured by tracking the optomotor response, a head-turning movement when the animal is presented with slowly moving vertical bars (Douglas et al., 2005). Normal C57BL/6 mice have a visual acuity between 0.38-0.40 cycles/degree (c/d). The DM-and Nog-treated eyes showed a significant reduction in visual acuity (DM=0.291±0.010 c/d; Nog=0.290±0.002 c/d), whereas the untreated and vehicle-treated eyes in these animals tracked normally (NT=0.386±0.001 c/d; vehicle=0.388±0.003 c/d). The results demonstrate that the inhibition of transient BMP signaling at the end of Müller glial development leads to persistent disruption in retinal structure and function.

### BMP signaling directly regulates Müller glial gene expression

**Fig. 6.**
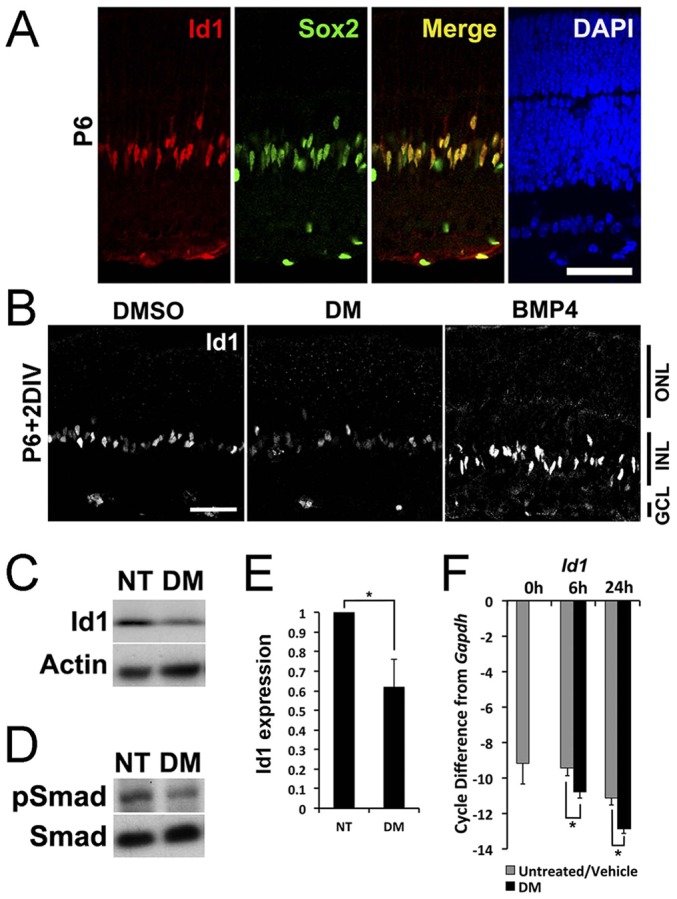
**Id1 and Id3 regulated by BMP signaling.** (A) At P6, Id1 (red) in Sox2+ Müller glia (green). Scale bar: 50 µm. (B) P6 retinal explants treated with factors for 2 days. Scale bar: 50 µm. (C-E) Intravitreal injection of DM at P6, retinas collected at P7. DM significantly decreased pSmad1/5/8 (D) and Id1 (C,E). Error bars are s.e.m. (*n*=6). *P<0.05 with paired *t*-test. (F) P6 retinal explants treated with DM for 0, 6, 24 h. qRT-PCR for *Id1* mRNA levels. Error bars are s.e.m. (*n*=4). *P<0.05, *t*-test.

To evaluate potential targets regulated by BMP signaling in Müller glia, we assayed by qRT-PCR the levels of several transcription factors previously shown to be involved in glial differentiation in explants treated with DM (Nelson et al., 2011). Previous reports have shown that Id1 and Id3 are both expressed in developing retinal progenitors at embryonic and neonatal stages of development (Du et al., 2010; Du and Yip, 2011; Mizeracka et al., 2013), but none have focused on this period of development. In addition, a previous report has shown that Id1 and Id3 are responsive to BMP signaling in the developing retina. To confirm Id1 expression in developing Müller glia, we co-labeled cells with Id1 and Sox2, another transcription factor expressed in Müller glia. We found that all Id1+ cells in the INL were also immunoreactive for Sox2 (Fig. 6A). The specificity of the anti-Id1 antibody was verified using tissues from Id1 knockout mice (supplementary material Fig. S7C,D). Immunohistochemistry, *in situ* hybridization and qRT-PCR showed a similar pattern of expression for both Id1 and Id3 in postnatal retina, consistent with previous findings (supplementary material Fig. S7A,B,E).

To determine whether the transient BMP signaling in the postnatal retina regulates Id1/3 expression, we inhibited the signal *in vitro* and *in vivo* and analyzed the effects. When DM was injected *in vivo* at P6, a significant reduction in pSmad1/5/8 and Id1 levels was observed 1 day after the injection (82.2±8.3% and 62.0±14.4% of NT, respectively) (Fig. 6E). In addition, treatment of retinal explants with DM significantly reduced *Id1* mRNA levels within 6 h of treatment (Fig. 6F), prior to the decline in the Müller glial markers *Rlbp1* or *Glul* (Fig. 3C). These data show that BMP signaling regulates Id1 expression in the retina during the stages of development, when BMP signaling is necessary for Müller glial development, and suggest that Id1 is part of the mechanism by which BMP signaling regulates the differentiation of these cells. However, it is unlikely that Id1 alone mediates the effects we observe on Müller glial development, as mice with a deletion in *Id1* do not show the same phenotype (supplementary material Fig. S8). As the highly related factor Id3 is expressed in a very similar pattern to Id1 during development and acts redundantly with Id1 in embryonic retina (Du and Yip, 2011), it might serve a redundant function in the postnatal retina as well.

Although the changes in Id1 precede the changes in *Rlbp1* or *Glul*, Id1 is not thought to be a transcriptional activator, and is thus not likely to directly drive expression of these genes. Thus, we also looked for additional transcription factors regulated by BMP signaling in the Müller glia. As noted above, we did not find significant reductions in *Sox9* in DM-treated retinas. Furthermore, we also did not find any changes in *Hes1*, *Nfib*, *Nfil3*, *Pou2f1* or *Mef2a* in DM-treated retinas (supplementary material Fig. S9A), suggesting that these transcription factors are not downstream of BMP signaling. Kuribayashi et al. (2014) recently reported that BMP induces an expression of the basic helix-loop-helix (bHLH) transcription factor Hey2 during early retinal development (E17), and proposed that Hey2 might affect Müller glial development (Kuribayashi et al., 2014). We did not observe a decrease in Hey2 expression when we inhibited BMP signaling in P6 retinal explants (supplementary material Fig. S9B); instead, we found a small increase in Hey2 expression following DM treatment.

**Fig. 7.**
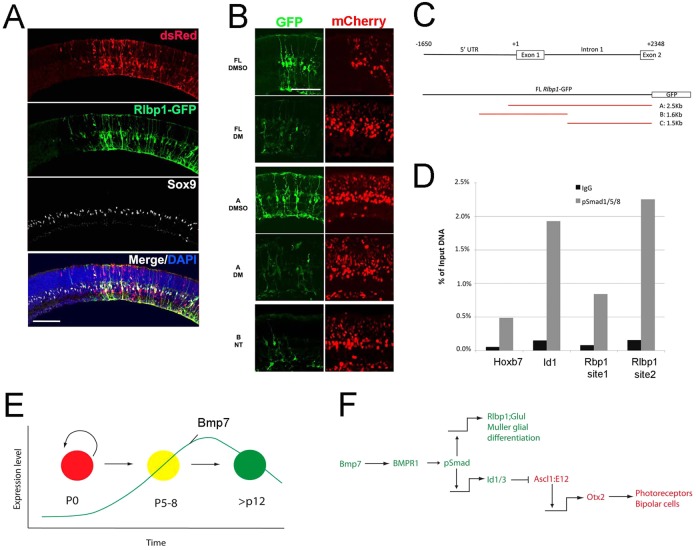
**Activation of *Rlbp1* promoter is regulated by transient BMP signaling during postnatal retinal development.** A plasmid containing *Rlbp1* promoter driving GFP expression (*Rlbp1-GFP*) was electroporated at P0 and retinas were cultured for 8 days (P0+8DIV). (A) *Rlbp1-GFP* FL (full-length) plasmid was electroporated at P0. At P0+8DIV, GFP+ cells colocalized with the Müller glial marker Sox9 (white). dsRed plasmid with a ubiquitous promoter was co-electroporated to locate transfected regions. (B) Indicated *Rlbp1-GFP* construct was electroporated. Expression of GFP was detectable in Müller glia from *Rlbp1-GFP* FL and A, whereas *Rlbp1-GFP* B and C did not show significant GFP expression (C not shown). DM at P0+3DIV: expression of *Rlbp1-GFP* FL and A showed significant reduction. Scale bars: 100 µm. (C) Specific fragments of the *Rlbp1-GFP* FL used (*Rlbp1-GFP* A-C). (D) Chromatin IP for pSmad on the *Id1* promoter (positive control) and two potential Smad consensus sites in the Rlbp1 first intron (arrows in B show genomic positions). Specific enrichment was found for the second site. (E,F) Model summary of results and potential mechanism for regulation of glial gene expression by BMP signaling.

As we were unable to find any other transcription factors that are regulated by BMP signaling in the postnatal Müller glia, we tested whether there might be a direct transcriptional regulation of pSmad1/5/8 on the *Rlbp1* promoter. The *Rlbp1* promoter consists of ∼1650 nucleotides 5′ to the first exon and including the first intron. This region drives Müller glia-specific expression in transgenic mice (Vazquez-Chona et al., 2009). We tested the *Rlbp1* promoter in retinal explant cultures (Fig. 7A,B) by electroporation at P0. dsRed or mCherry constructs were co-electroporated in order to identify the transfected regions. The Rlbp1-GFP co-labels with the Müller glial marker Sox9 and the cells have the morphology of Müller glia (Fig. 7A). We tested whether the *Rlbp1* promoter was responsive to BMP signaling by treating electroporated explants with DM, starting 3 days after electroporation for 5 days. GFP expression in the electroporated cells was consistently decreased in DM-treated explants (Fig. 7B). Smaller fragments of the *Rlbp1* promoter were also electroporated to narrow the region of BMP responsiveness (Fig. 7B,C). Construct A, which included the first exon and intron, but only a short sequence upstream of the first exon, drove GFP expression specifically in the Müller glia, which was also decreased with DM. Both the first exon and first intron were also required for Müller glial expression, as constructs B and C did not show consistent or high levels of GFP in the Müller glia (Fig. 7B,C; construct C not shown).

The region encompassed by the 2.5 kb A fragment has potential Smad consensus sites. To test for a direct interaction between Smad and the *Rlbp1* promoter or nearby enhancer sequences, we carried out chromatin IP for Smad1/5/8 in P6 retina. Id1 has previously been shown to be a direct target for BMP signaling and we used the *Id1* promoter containing a previously validated Smad consensus site as a positive control, whereas the *Hoxb7* promoter served as a negative control. We found that Id1 and one of the Smad sites in the *Rlbp1* first intron showed consistent enrichment for pSmad1/5/8 in the chromatin IP (Fig. 7D). Thus, the effects of BMP signaling on Müller glial gene expression, at least for *Rlbp1*, might function directly through pSmad1/5/8 binding to an enhancer in the first intron of the *Rlbp1* gene.

## DISCUSSION

In this study, we show a transient BMP-mediated Smad1/5/8 phosphorylation in the developing Müller glia at the end of the first postnatal week in the mouse retina. Inhibition of this transient activation significantly reduces expression of glial genes, and many of the Müller glia fail to repress the neural transcription factor Otx2. Although the number of Müller glia is not altered, inhibition of BMP signaling permanently disrupts retinal morphology and impairs retinal function. Together, we conclude that the transient activation of BMP-Smad1/5/8 signaling in Müller glia is essential for stabilizing their glial phenotype during retinal development.

During embryonic ocular development, BMP signaling has been shown to regulate the patterning of the optic vesicle, lens development, progenitor proliferation and differentiation, axon guidance and neuronal survival (Dudley and Robertson, 1997; Furuta and Hogan, 1998; Wawersik et al., 1999; Trousse et al., 2001; Zhao et al., 2002; Yang, 2004; Plas et al., 2008). BMP2, BMP4 and BMP7 are potential ligands for these processes (Du et al., 2010). These BMP ligands are expressed from embryonic day (E) 13.5 to P1, although only BMP7 was detected in the adult retina by western blot (Du et al., 2010). Our *in situ* hybridization localized *Bmp7* expression to the inner retinal cells, including amacrine cells, horizontal cells, bipolar cells and ganglion cells; however, only relatively small numbers of each of these cells types expressed this ligand at any age. RPE and ciliary epithelium express much higher levels of BMP ligands than the retina in the postnatal eyes, and could also be the source for these ligands. In previous studies, Bmpr1 expression was detected at low levels in the postnatal and adult retinas (Du et al., 2010), and both *Bmpr1b* and *BmprII* were shown to be localized in the INL at P7 by *in situ* hybridization (Liu et al., 2003). Our results demonstrate that *Bmpr1a* and *Bmpr1b* are expressed in the INL at P6, corresponding to the period of transient BMP signaling activation in the Müller glia; however, whereas Bmpr1b is expressed ventrally, Bmpr1a is expressed throughout the retina. Throughout postnatal development, there was no significant change in the expression levels of BMP ligands, either in the retinas or RPE, and at least one type 1 receptor is present from P0 to P12. Thus, the transient activation of BMP signaling in the INL must be explained by some co-regulator that we have not identified yet. We did not detect pSmad1/5/8 in the retinas after P9, and total Smad1/5/8 levels also declined after P9. The loss of Smad1/5/8 expression might thus explain the end of transient activation of BMP signaling in the developing Müller glia.

Our data indicate that the transient BMP-Smad1/5/8 signaling in the retina is essential for Müller glial differentiation. When BMP signaling was blocked, there was a significant decrease in the levels of glial gene expression, including Cralbp, GS and Id1, both *in vitro* and *in vivo*. In addition, inhibition of BMP signaling leads to the expression of Otx2 (a neural transcription factor that is normally expressed in the photoreceptors and bipolar cells) in the Sox9+ Müller glia. The number of Sox9+ cells is not significantly different after DM treatment, both *in vitro* and *in vivo*, suggesting that inhibition of BMP signaling induces the Otx2 expression in the Müller glia rather than the induction of Sox9 expression in the Otx2+ bipolar cells, and that Müller glial fate choice is not regulated by BMP signaling at this stage of development. This is further supported by the fact that Ascl1 lineage tracing shows the same proportions of Müller glia and bipolar cells in DM-treated retinas as in controls. These results are in contrast to the conclusions reached by two earlier studies that showed effects on BMP inhibition on Müller glial fate. Huillard et al. (2005) found that, in chick embryos, expression of the BMP antagonist Drm (Grem1 – Mouse Genome Informatics) led to a loss in Müller glial cells in the infected region. However, the presence of Müller glia was assessed in this study by QR1 (Nqo1 – Mouse Genome Informatics) labeling (an extracellular matrix molecule specifically expressed in Müller glia); it is possible that the Müller glia were present but downregulated this differentiation marker, similar to GS or Cralbp in the present study. More recently, Kuribayashi et al. found that overexpression of BMPR1a/b in E17 retinas led to an increase in the number of Müller glia, bipolar cells and amacrine cells, and a decrease in photoreceptors *in vitro*, whereas expression of dominant negative BMPR1a/b at E17 decreased the number of Müller glia and bipolar cells (Kuribayashi et al., 2014). We did not observe any difference in the number of Müller glia, bipolar cells, amacrine cells and ganglion cells when BMP-Smad1/5/8 was inhibited *in vivo* at P6. The difference in these results might be due to the difference in the timing of BMP inhibition (E17 versus P6) and/or differences between *in vitro* (Kuribayashi et al., 2014) and *in vivo* (present study) conditions. It is also interesting that we found Id1/3 increased by BMP signaling (Fig. 6) at P6. Both Id1 and Id3 are known to promote glial fates from progenitors (Mizeracka et al., 2013); however, by P6, the progenitors are apparently committed to glial or neuronal fates.

Although BMP signaling at the end of the first postnatal week does not affect glial cell fate, BMP-mediated regulation of glial maturation is clearly essential for proper development of the Müller glia, as, without the transient BMP signaling, Müller glia fail to support normal retinal architecture and function. It has been well demonstrated that the health of Müller glia is essential for retinal development and function (Bringmann et al., 2006). Müller glia are the predominant retinal glial cell type, spanning its entire thickness, and they maintain the homeostasis of water, ions and neurotransmitters in the retina. As Müller glia interact with all types of retinal neurons, impairment in their differentiation would disrupt retinal morphology as well as function. A study by Shen et al*.* has demonstrated that conditional ablation of Müller glia in the adult retina induces photoreceptor cell death and vascular abnormalities (Shen et al., 2012). Interestingly, the retinas with selective Müller glial ablation show ‘mushroom-like’ bulges of photoreceptors similar to those observed in this study. It is also interesting that the OLM is disrupted in the DM- or Nog-treated retinas (as assayed by ZO-1 staining). Mutations in several different genes coding for OLM proteins produce similar phenotypes to that which we have observed after inhibition of BMP signaling at P6 (Cho et al., 2012; Alves et al., 2013). Although we do not know whether the OLM genes are directly regulated by BMP signaling in this system, there is evidence in other tissues that BMP can regulate the expression of components of the tight junctional complex (Saitoh et al., 2013).

There are several potential mechanisms by which the transient BMP-Smad1/5/8 signaling might promote Müller glial differentiation. Several transcription factors are known to be important for Müller glial development, such as Sox9, Hes1 and Hes5 (Furukawa et al., 2000; Hojo et al., 2000; Poche et al., 2008; Nelson et al., 2011). Both Sox9 and Hes5 were reduced in their expression after BMP inhibition, but this took several days, whereas the expression of Cralbp and GS occurred with a more rapid time course. Analysis of the *Rlbp1* promoter provides evidence for a more direct interaction. There are Smad1/5/8 binding sites in the *Rlbp1* and *Glul cis*-regulatory regions, including the 2.5 kb fragment of the *Rlbp1* promoter that is BMP responsive (Fig. 7), and one of these Smad consensus sites is enriched for pSmad1/5/8 binding by chromatin IP at P6. It is also interesting that the inhibition of BMP signaling at P6 has lasting effects on glial morphology. This appears to be due to a transient requirement for this signal for the glial cells to properly reach a mature state, as inhibition of BMP signaling after this crucial period, either at P10 (this study) or in adult retina (Ueki and Reh, 2012), does not cause this phenotype.

In addition to this direct effect, it is possible that BMP-Smad1/5/8 regulates glial differentiation via Id1 and Id3. *Id1* mRNA is significantly decreased by 6 h after DM treatment, which is observed prior to the decline in *Rlbp1* and *Glul* mRNA levels. Previous evidence has shown that BMP regulates expression of Id1 and Id3 in many systems, including the retina (Korchynskyi and ten Dijke, 2002; Lopez-Rovira et al., 2002; Du et al., 2010; Ueki and Reh, 2012). Our data demonstrate that, in the postnatal retinas, Id1 is expressed in the Müller glia at the time of transient BMP-Smad1/5/8 activation, and that Id1 expression is regulated by BMP signaling in the Müller glia both *in vitro* and *in vivo*. Previous studies have shown that Id1 and Id3 are important for retinal development: double-knockout mice display smaller retinas compared with WT at E13.5 (Du and Yip, 2011), and overexpression of Id1 and Id3 in the retina starting at P0 increases the number of cells that resemble retinal progenitors or Müller glia in the mature retina (after P14) (Mizeracka et al., 2013). The DM-induced decrease in Id1 expression that we observe in the Müller glia at P6 does not affect the total number of Müller glia in the retina, but this might result from the difference in the timing of the experiments. We propose that BMP signaling maintains Id1/3 levels in the Müller glia as they transition from progenitors to glia (Fig. 7E,F). Id1/3 in turn inhibit the actions of proneural transcription factors, such as Ascl1 and Olig2 (Benezra et al., 1990; Samanta and Kessler, 2004; Vinals et al., 2004), preventing them from expressing neural determination factors, including Otx2, and thus stabilizing the glial fate (Fig. 7E,F). Interestingly, our previous study showed that, when Müller glia are forced to express the proneural transcription factor *Ascl1 in vitro*, they lose the glial phenotype and display progenitor-like properties (Pollak et al., 2013). Ascl1-overexpressing Müller glia show decreases in glial gene expression, whereas neural and progenitor genes, including *Otx2*, are induced. Thus, the regulation of key transcription factors in Müller glia is essential for their phenotype, and the transient BMP signaling might contribute to this process during postnatal development.

BMP signaling is known to promote astrogliogenesis in the CNS (Mabie et al., 1997; Grinspan et al., 2000; See et al., 2004, 2007; Gomes et al., 2003). This fate change is potentially mediated through BMP-induced upregulation of Id1/3 and Hes5, which in turn inhibit Ascl1 activity (Nakashima et al., 2001). Cortical astrogliogenesis by BMP-Smad1/5/8 activation is potentiated by the activation of LIF/gp130/STAT3 signaling (Nakashima et al., 1999a,b; Fukuda et al., 2007). These effects on cell fate are typically assayed using genes expressed in the differentiated glia, such as Gfap or S100β. If BMP also promotes astrocyte maturation, as it does for Müller glia, BMP might have a role later in astrocyte development than currently appreciated. Our results show that BMP-Smad1/5/8 signaling is required for Müller glial differentiation, and for proper retinal development and function by maintaining glial gene expression and repressing neural gene expression. Due to the similarities of Müller glia with astrocytes, and the demonstrated role for BMP in astrocyte development, a similar transient BMP signal might stabilize the differentiation of glia in other regions of the CNS.

## MATERIALS AND METHODS

### Animals

C57BL6 mice were used at indicated age unless otherwise stated. For the lineage-tracing study, *Ascl1^CreERT2^* knock-in mice (Kim et al., 2011) and mTmG reporter mice (Jackson Laboratory, stock 007576) (Muzumdar et al., 2007) were used. All mice were housed at the University of Washington; protocols were approved by the University of Washington Institutional Animal Care and Use Committee.

### Retinal explant cultures

Retinas were explanted at indicated ages and cultured as described previously (Ueki et al., 2012). Media and factors were replaced every other day at the following concentrations: BMP4, 100 ng/ml; DM, 5 µM; DAPT, 10 µM; EGF, 100 ng/ml.

### Immunohistochemistry (IHC)

Eyecups or retinal explants were fixed in 2% paraformaldehyde for 45 min at room temperature. After a single PBS wash, samples were cryoprotected in 30% sucrose overnight at 4°C. Eyecups or explants were embedded in OCT compound (Sakura Finetek) and sectioned at 12-14 µm. For IHC analysis, sections were blocked with 10% normal horse serum (Vector Labs) containing 0.5% Triton X-100 for 1 h at room temperature, then primary antibodies were applied and incubated at 4°C overnight. Primary antibodies used are listed in supplementary material Table S1. Sections were washed three times with PBS, and incubated with secondary antibodies (Life Technologies) and DAPI (Sigma) for 1 h at room temperature. Sections were coverslipped and confocal microscopy was performed using an Olympus FluoView FV1000.

### *In situ* hybridization

Digoxigenin-labeled probes were transcribed *in vitro* from linearized cDNA clones. The cDNAs used were: Bmp4 and Bmp7 (gifts from Dr Brigid Hogan, Duke University, Durham, NC, USA), Bmpr1a (OpenBioSystems, CloneID: 5364272), Bmpr1b (OpenBioSystems, CloneID: 30357130) and Bmp7 (OpenBioSystems, CloneID: 4218495). The hybridization was carried out according to Etchevers et al. (2001). Signals were visualized using an anti-digoxigenin alkaline phosphatase-conjugated secondary antibody (Roche).

### Western blot

Retinas were homogenized in lysis buffer. Equal amounts of protein samples were loaded in each well of a 4-20% SDS gel (Bio-Rad) and standard WB procedures were performed with HRP-conjugated secondary antibodies (Bio-Rad); signals were visualized on x-ray film using SuperSignal West Dura Extended Duration Substrate (Thermo Scientific) and quantified using ImageJ software (NIH); β-actin was used as a loading control. Primary antibodies are listed in supplementary material Table S1.

### Quantitative RT-PCR (qRT-PCR)

For RPE isolation, enucleated eyes were incubated in 200 µg/ml proteinase K (Sigma) for 10 min at 37°C to partially digest the sclera. Following the incubation, the cornea and sclera were carefully removed, and the retina and RPE were separated. The RPEs and retinas from 12-18 eyes were pooled in one tube and RNA isolation was performed using TRIzol (Invitrogen). cDNA was created using the iScript cDNA synthesis kit (Bio-Rad) and qPCR was performed using SSO Fast reagent (Bio-Rad). Primers used are listed in supplementary material Table S2.

### Intravitreal injections

Mice at the indicated ages were anesthetized with isoflurane, and 1 µl of vehicle (DMSO:PBS=1:1), dorsomorphin (DM) (5 µM) (Tocris Bioscience) or Nog (100 µg/µl) (R&D Systems) was injected intravitreally using a Hamilton syringe with a 32-gauge needle (Hamilton). The other eye of each animal was either uninjected or injected with vehicle.

### Ascl1 lineage tracing

*Ascl1^CreERT2^* knock-in mice (Kim et al., 2011) were crossed with mTmG reporter mice (Jackson Laboratory, stock 007576) (Muzumdar et al., 2007). Pups were injected with 0.5 mg tamoxifen (Sigma) in 50 µl corn oil intraperitoneally at P4 to label the lineage of Ascl1+ retinal progenitors at the time of injection (rod photoreceptors, bipolar cells and Müller glia) with GFP. DM was injected intravitreally at P6 in one eye and the other eye was uninjected as a control. Eyes were collected at P21. GFP+ rod photoreceptors (in the ONL), bipolar cells (Otx2+ in the INL) and Müller glia (Otx2− in the INL) were blindly counted from random images of the central retina.

### Optomotor response measurement

Intravitreal injections of vehicle, DM or Nog were performed at P6. Some eyes were left uninjected (NT) as controls. At P28, the visual acuity of each eye was measured by detecting the tracking movement for a random spatial frequency grating with 100% contrast using the OptoMotory device (CerebralMechanics).

### *In vitro* electroporation of *Rlbp1*-GFP plasmid

P0 retinas were isolated and electroporated with a plasmid containing the *Rlbp1* promoter driving GFP expression (*Rlbp1-GFP*) (Vazquez-Chona et al., 2009). The *Rlbp1* promoter was cleaved into shorter lengths using InFusion cloning (Clonetech). A construct encoding dsRed or mCherry was co-electroporated in order to detect transfected regions of the retina. After the electroporation, the retinas were cultured as explants. DM or vehicle was added, starting 3 days after the electroporation (P0+3DIV) for 5 days. At P0+8DIV, explants were collected for histological analysis.

### Chromatin immunoprecipitation (ChIP)

P6 retinas were dissociated to single cells with papain, resuspended in PBS at 20 million cells per ml, followed by fixation with 1% formaldehyde for 10 min, rotating at room temperature. Sheering of chromatin was performed with a Fisher Scientific probe sonicator with settings: 10 pulses of 100 J, 45 Amplitude, 45 s offset, 4°C. Immunoprecipitation was performed with 25 μl anti-rabbit IgG magnetic beads (Invitrogen, #112-03D) and 5 μl rabbit anti-pSmad1/5/8 (Cell Signaling, #9511S) antibody or 3 μg rabbit IgG (R&D Systems, AB-105-C) against chromatin from 2×10^5^ cells per IP according to LowCell # ChIP Kit (Diagenode). ChIP qPCR primers are listed in supplementary material Table S3.

## Supplementary Material

Supplementary Material
