# Supplementary material for: A transient wave of BMP signaling in the retina is necessary for Müller glial differentiation
Source: Development. 2015 Feb 1;142(3):533–43. doi: 10.1242/dev.118745 (PMC4302996; doi:10.1242/dev.118745)
Supplement: Supplementary Material [file supp_142_3_533__index.html]

Supplementary Material 

# A transient wave of BMP signaling in the retina is necessary for Müller glial differentiation

## DEV118745 Supplementary Material

**Files in this Data Supplement:**

- Supplementary Material
