# Supplementary material for: A transient wave of BMP signaling in the retina is necessary for Müller glial differentiation
Source: Development. 2015 Feb 1;142(3):533–43. doi: 10.1242/dev.118745 (PMC4302996; doi:10.1242/dev.118745)
Supplement: Supplementary Material [file supp_142.3.533_DEV118745supp.pdf]

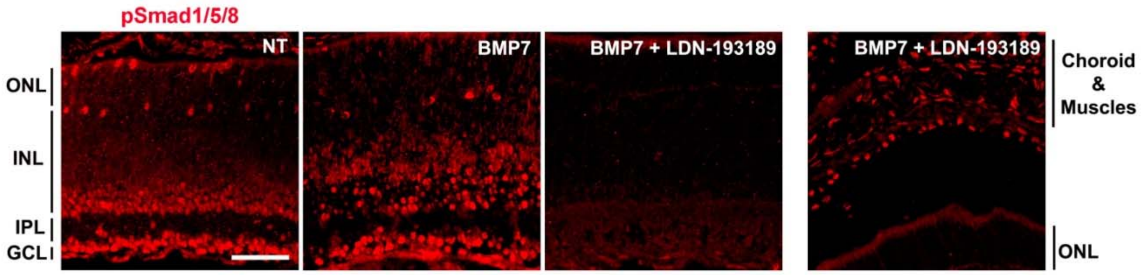

Supplemental Figure 1. pSmad1/5/8 antibody detected specific signal in IHC. Specificity of pSmad1/5/8 used in the study was evaluated using a BMP signaling inhibitor, LDN-193189. Intravitreal injection of an agonist BMP7 and/or LDN-193189 was performed at P5, and retinas were harvested at P6 for pSmad1/5/8 IHC. Untreated retina (NT) shows pSmad1/5/8 immunoreactivity (red) mainly in differentiated cells in the GCL and lower INL. Injection of BMP7 did not significantly change distribution of pSmad1/5/8+ cells, suggesting that Smad1/5/8 was activated at maximum level with endogenous ligands. Injection of BMP7 along with LDN-193189 completely abolished pSmad1/5/8 immunoreactivity, demonstrating the specificity of the pSmad1/5/8 antibody. In contrast, extraocular tissues (such as choroid and extraocular muscles) that were not affected by the intravitreal injection of LDN-193189 retained the pSmad1/5/8 signal. GCL, ganglion cell layer; IPL, inner plexiform layer; INL, inner nuclear layer; ONL, outer nuclear layer. Scale bar: 50 µm.

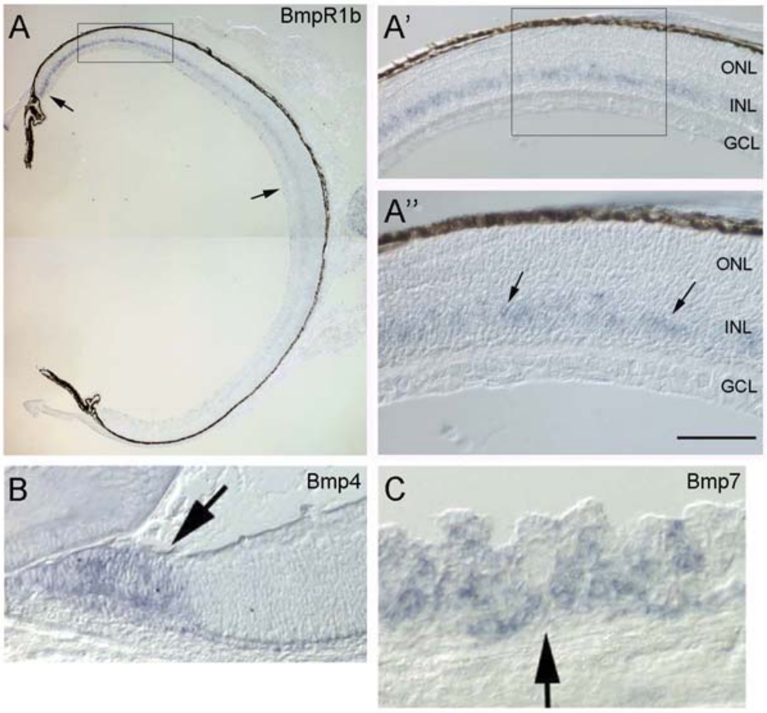

Supplemental Figure 2. *In situ* hybridization for Bmpr1b (A-A'') at P6, Bmp4 (B) in the ciliary body at P0 and (C) Bmp7 in the ciliary body at P6. Expression of Bmpr1b is in the INL, consistent with the pSmad1/5/8 signaling at this age; however, Bmpr1b shows a distinct dorsal-ventral gradient in expression, whereas pSmad1/5/8 is present across the entire retina at this age.

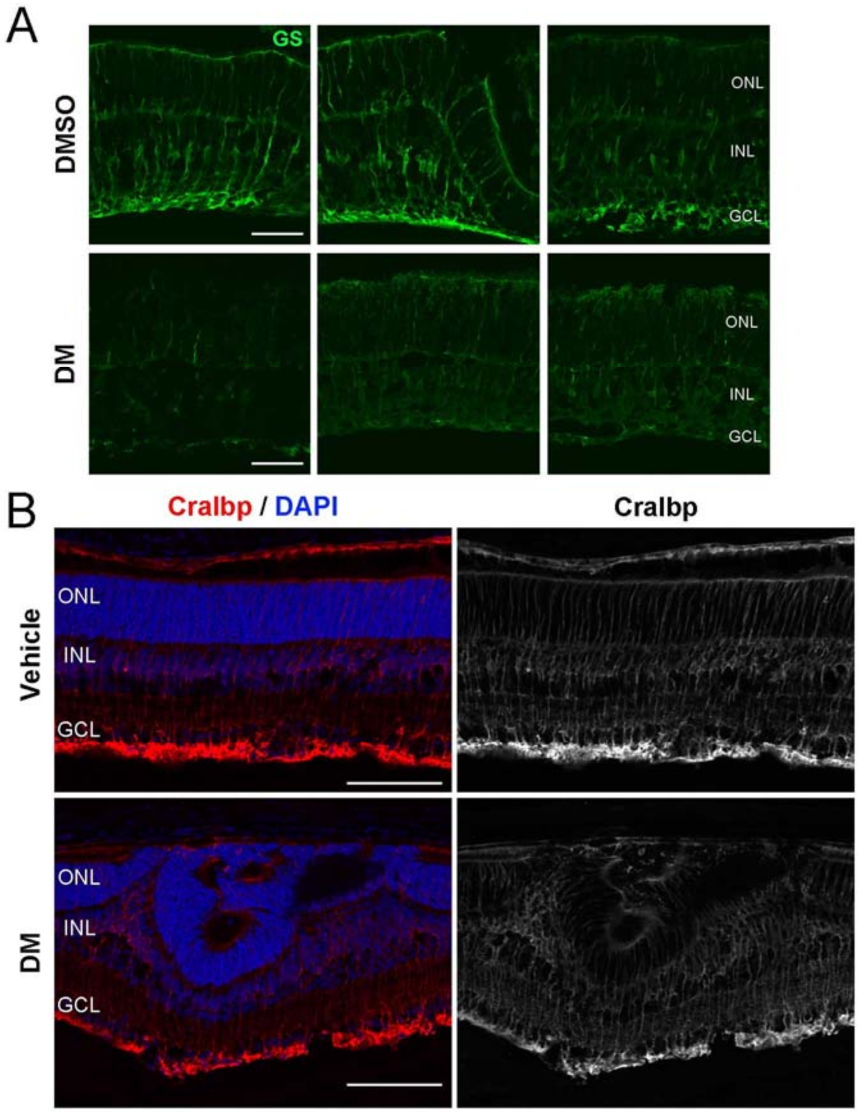

Supplemental Figure 3. Inhibition of BMP signaling causes a reduction in the labeling for GS (A) and Cralbp (B) in representative sections. (A) DMSO or DM treated retinal explants cultured for 6 DIV and labeled for glutamine synthetase (green). (B) DM or untreated retinas at P6 collected at P14 show a reduction in Cralbp labeling, particularly evident in the rosettes.

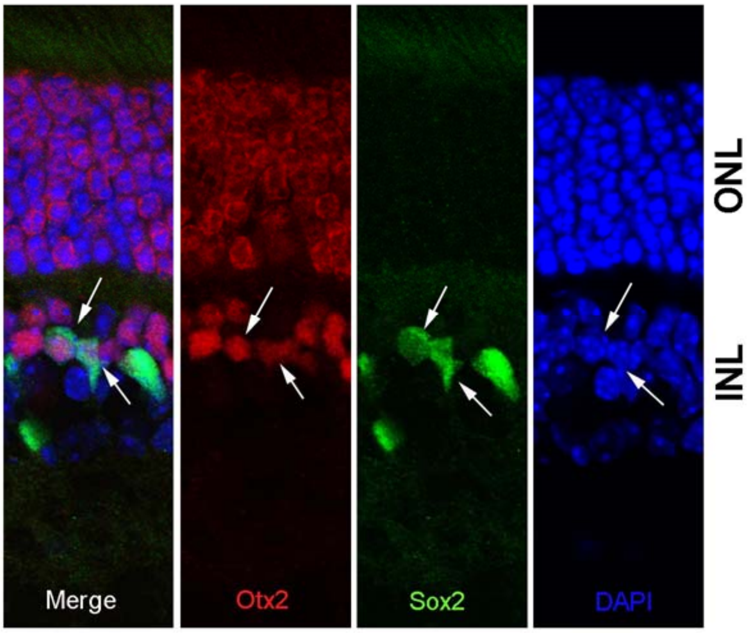

Supplemental Figure 4. Otx2 and Sox2 labeling of retina treated with DM at P6 and collected at P21. Image shows single 1 μm confocal section. Arrows point to double labeled cells.

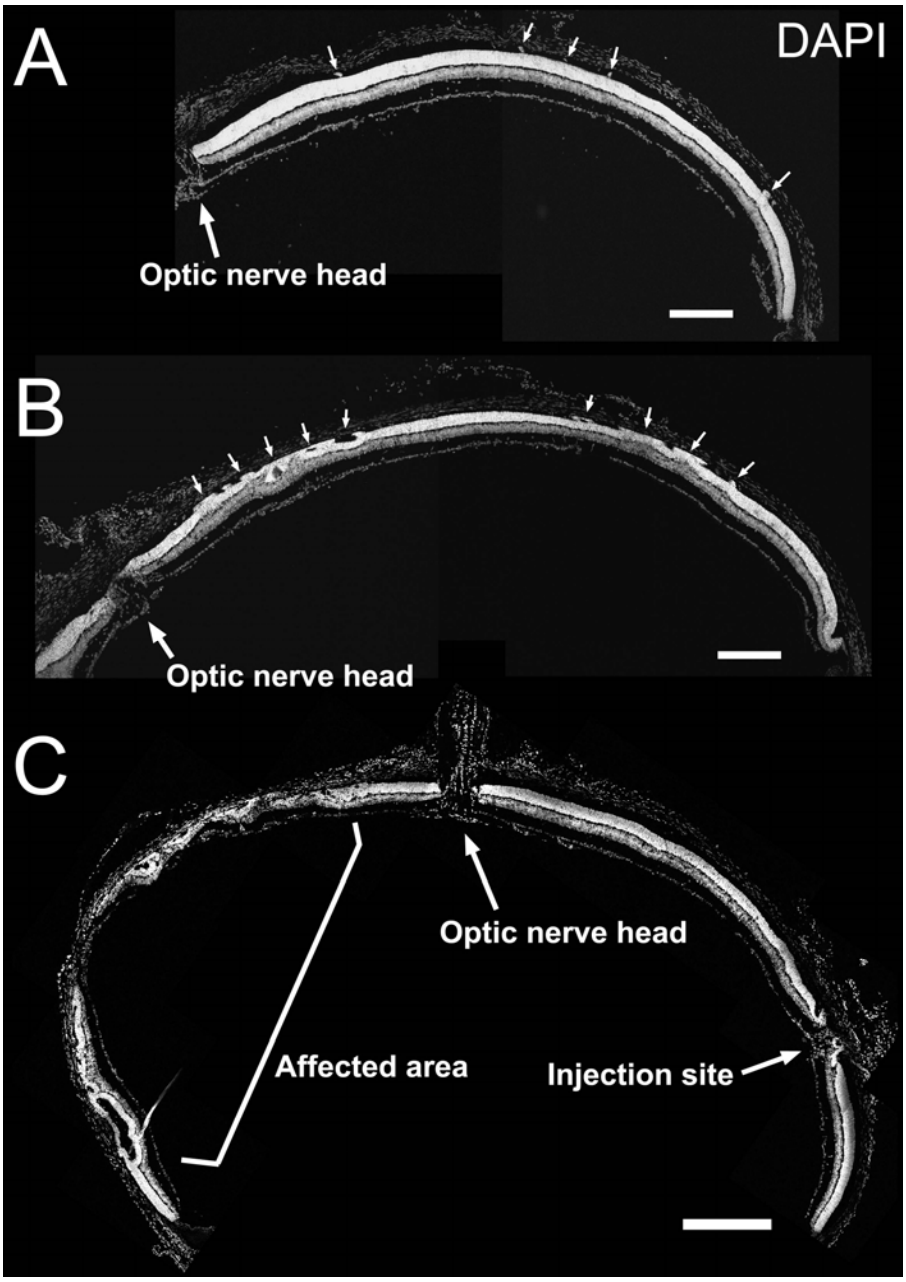

Supplemental Figure 5. Inhibition of transient BMP-Smad1/5/8 signaling during postnatal development causes persistent panretinal disruption in retinal morphology. DM or Noggin was injected at P6, eyes were collected at P21 and nuclei were labeled with DAPI. Representative images of the DM-injected retinal hemisphere are shown. (A) Multiple bulges of photoreceptors (arrows) are observed in the retinas with milder morphological disruption. (B) Many of the DM or Noggin treated retinas showed multiple rossette structures and photoreceptor bulges (arrows) across the retina. (C) In the most severe cases, the entire retina had severe morphological disruption with rosettes and thinning of the retina. Scale bars in A-B: 100 µm; in C: 500 µm.

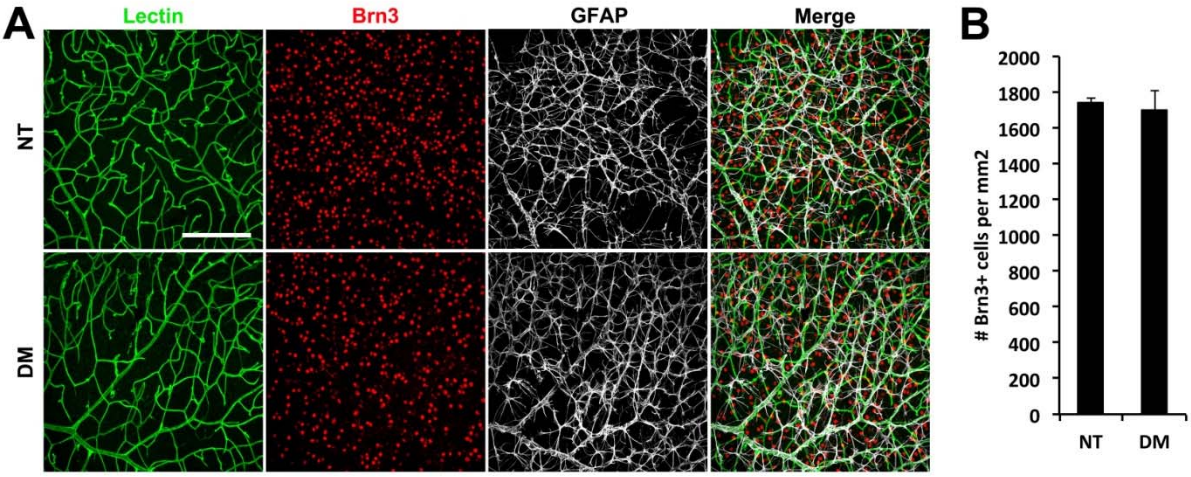

Supplemental Figure 6. Inhibition of transient BMP-Smad1/5/8 signaling during postnatal development does not cause apparent changes in the ganglion cells, astrocytes, or retinal vasculature. DM was injected at P6 and eyes were collected at P21. (A) The retinal vasculature, ganglion cells and astrocytes were visualized in retinal flatmounts with lectin (green), Brn3 (red) and GFAP (white) antibodies, respectively. There were no apparent phenotypes in these cell types in DM treated retinas. (B) The number of Brn3+ ganglion cells was counted in the retinal flatmounts. There was no significant difference in ganglion cell numbers between NT and DM (1743.0±24.2 and 1699.7±108.2 Brn3+ cells per mm^2, respectively). n=5.

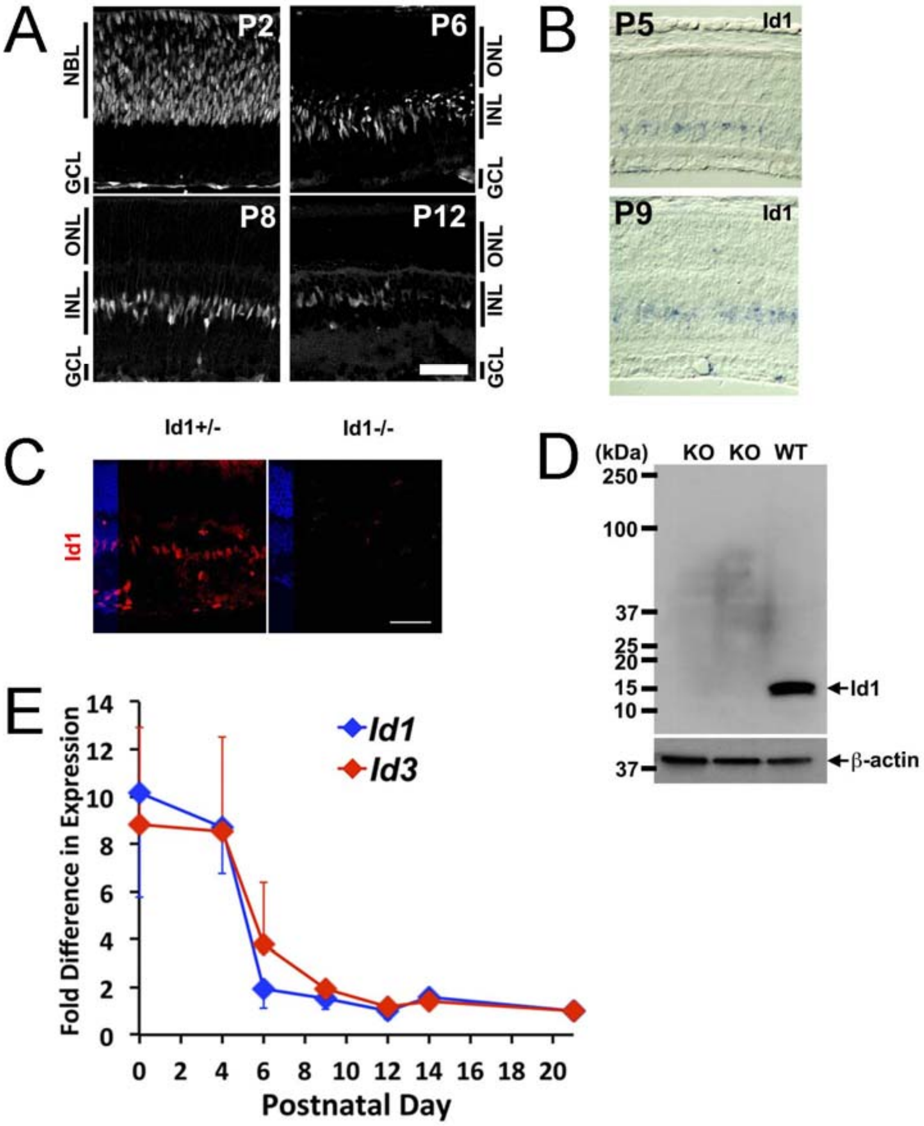

Supplemental Figure 7. Id1 and/or Id3 may be part of the mechanism by which BMP signaling regulates Müller glial differentiation. (A) Id1 expression during postnatal retinal development was examined by IHC. Id1 was expressed in progenitor cells (P2-4, NBL) and Müller glia (P6-P10). Id1 expression is undetectable by P14 (not shown). (B) *In situ* hybridization for *Id1* correlated well with IHC localization shown in A. At P9, expression is restricted to the Müller glial nuclei in the INL. NBL, neuroblastic layer; GCL, ganglion cell layer; INL, inner nuclear layer; ONL, outer nuclear layer. (C) Specificity of the Id1 antibody was tested for IHC. Id1 immunoreactivity was not detected in Id1$^{-/-}$ retina. Scale bar: 50 µm. (D) Spleen samples of adult Id1 KO and WT mice were collected, and Western blot was performed. While WT sample showed single band at 17KDa (expected size for Id1), KO samples did not show any band. Actin was used as a loading control. (E) qRT-PCR analysis showed rapid decline in *Id1* and *Id3* mRNA expression in the retina during postnatal development. Error bars are SEM. (n=3).

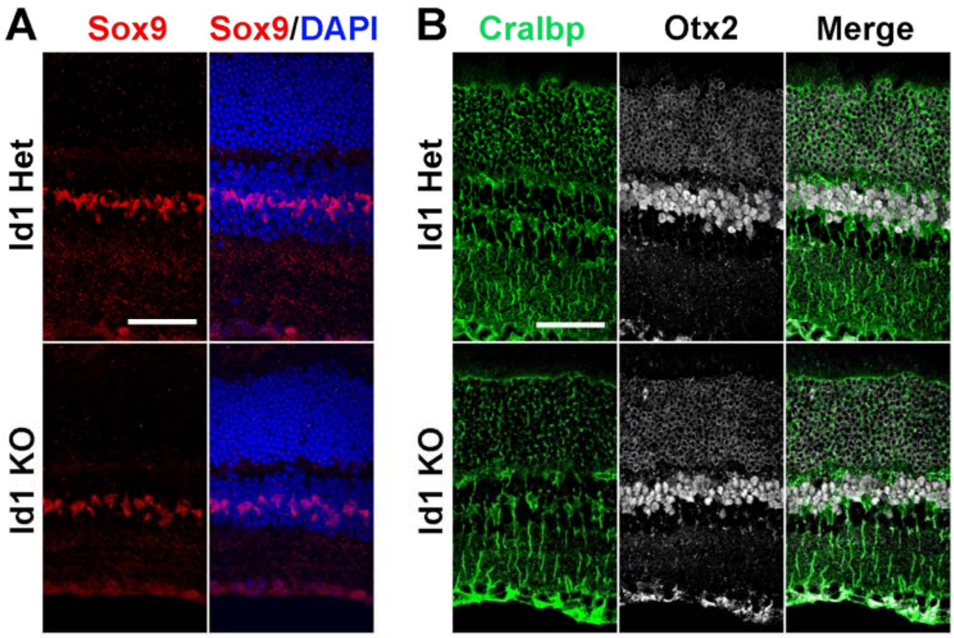

Supplemental Figure 8. Müller glia in Id1KO retinas were normal. Retinas from 13-month old Id1 Het and KO littermates were collected, and immunohistochemistry for Müller glial markers were performed. (A) Sox9 is a nuclear marker of Müller glia. The number and localization of Sox9+ cells (red) were normal in Id1KO retinas. Nuclear staining with DAPI (blue) showed normal gross morphology of Id1KO retinas. (B) A Müller glial marker Cralbp (green) delineated normal morphology of Müller glia in Id1KO retinas. Müller glial nuclei did not show any colocalization with Otx2 (white; bipolar and photoreceptor cell marker). Scale bars: 50 μm.

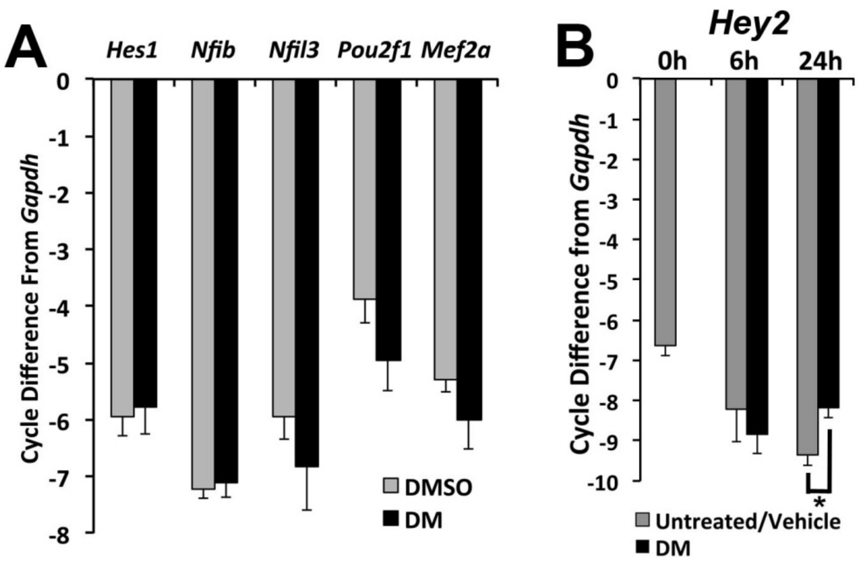

Supplemental Figure 9. Inhibition of BMP signaling with DM did not alter expression levels of many Müller glial transcription factors. (A) Retinas were explanted at P3 and BMP signaling was inhibited by DM for 5 days *in vitro* (P3+5DIV; prior to its transient activation period). mRNA levels of transcription factors that might affect Müller glial development were measured by qRT-PCR. There was no significant difference in *Hes1*, *Nfib*, *Nfil3*, *Pou2f1* and *Mef2a* expression between NT vehicle and DM treated explants. Error bars are SEM (n=3). (B) P6 retinal explants were treated with vehicle or DM for 0, 6, and 24hrs and collected for *Hey2* qRT-PCR. DM treatment did not alter expression of *Hey2* by 6 hrs. Increase in *Hey2* expression was detected in DM treated explants compared to NT by 24 hrs. Error bars are SEM (n=at least 3 per group). *$p < 0.05$ with t-test.

Supplemental Table S1. Antibodies.

**Protein**	**Vendor**	**Host**	**Application and Dilution**
pSmad1/5/8	Cell Signaling Technology	Rabbit	IHC 1:100<br>WB 1:2500
Smad1/5/8	Santa Cruz Biotechnology	Rabbit	WB 1:2000
Id1	BIOCHECK	Rabbit	IHC 1:1000<br>WB 1:10000
Cralbp	abcam	Mouse	WB 1:10000
Cralbp	gift from Dr. Jack Saari	Rabbit	IHC 1:750
Glutamine synthetase	Millipore	Mouse	WB 1:5000
Sox9	Millipore	Rabbit	IHC 1:1000<br>WB 1:2000
Sox2	Santa Cruz Biotechnology	Goat	IHC 1:200<br>WB 1:2000
GFAP	DAKO	Rabbit	IHC 1:500
Otx2	R&D Systems	Goat/Biotinylated	IHC 1:250
PKC	Sigma	Mouse	IHC 1:250
AP2a	Developmental Systems Hybridoma Bank	Mouse	IHC 1:250
Brn3	Santa Cruz Biotechnology	Goat	IHC 1:100
Sib4 (lectin)	Vector Labs	Biotinylated	IHC 1:250
β–actin	abcam	Mouse	WB 1:20000

Supplemental Table S2. qRT-PCR primers.

Gene	Forward	Reverse
Gapdh	GGCATTGCTCTCAATGACAA	CTTGCTCAGTGTCCTTGCTG
Bmp2	GGGACCCGCTGTCTTCTAGT	TCAACTCAAATTCGCTGAGGAC
Bmp4	GACTTCGAGGCGACACTTCTA	GCCGGTAAAGATCCCTCATGTAA
Bmp5	TTACTTAGGGGTATTGTGGGCT	CCGTCTCTCATGGTTCCGTAG
Bmp6	AGAAGCGGGAGATGCAAAAGG	GACAGGGCGTTGTAGAGATCC
Bmp7	ACGGACAGGGCTTCTCCTAC	ATGGTGGTATCGAGGGTGGAA
Bmp8a	ACATGCAGCGTGAAATCCTG	GCGTGGTATAGGTCCAACATGA
Bmp8b	CCGGGACTCCTATGGCTACT	CATCCGTCATGGCACGGTA
Bmp10	ATGGGGTCTCTGGTTCTGC	CAATACCATCTTGCTCCGTGAA
Bmp15	TCCTTGCTGACGACCCTACAT	TACCTCAGGGGATAGCCTTGG
Gdf2 (Bmp9)	CAGAACTGGGAACAAGCATCC	GCCGCTGAGGTTTAGGCTG
Gdf5	TGCCCCTGACTTAGGACAGAG	ACACCATAGATATGACCCCCTG
Gdf6	TATCGCGCCCCTAGAGTACG	ATGCTAATGGGAGTCAGTTTGG
Gdf7 (Bmp12)	GAGCTTCCTGTTCGACGTATC	CAGGCAGAACTTGCGGGAG
Amh	CCACACCTCTCTCCACTGGTA	GGCACAAAGGTTCAGGGGG
Rlbp1	ACCAAGGATCATGGTCCTGTC	CCTGTGCCTGTACCAGCTC
Glul	GTTCCCACTTGAACAAAGGCA	ACCCAGATATACATGGCTTGGA
Id1	TACGACATGAACGGCTGCTACTCA	TTACATGCTGCAGGATCTCCACCT
Id3	CTGTCGGAACGTAGCCTGG	GTGGTTCATGTCGTCCAAGAG
Hes1	TCAACACGACACCGGACAAAC	ATGCCGGGAGCTATCTTTCTT
Nfib	TGAGGCAGCTTCACCTACAG	AGGATGGGTCTCTTGGGCTTA
Nfil3	TATTGGGAGAAACGGCGGAAA	AGCCTTGGATGTCTGGTAGTC
Pou2f1	AGCTGGGACAAGTTTACAGGC	TCCCGACTCTTCACTGGATTTA
Mef2a	CAGGTGGTGGCAGTCTTGG	TGCTTATCCTTTGGGCATTCAA
Hey2	TGAGAAGACTAGTGCCAACAGC	TGGGCATCAAAGTAGCCTTTA

Supplemental Table S3. ChIP qPCR primers

Primer	Sequence
pRlbp Smad site 2 L	TCGCCTTTCCTAGTTTCCTCT
pRlbp Smad site 2 R	CCCCAGAGCTTGTGTCTCTA
pRlbp Smad site 1 L	CTCCTGTGAGCCTGACTGAA
pRlbp Smad site 1 R	AATTGTTCTGGAGCTCGGGA
pHoxb7 L	TTCAATCCCTGCGTCTCTCT
pHoxb7 R	CTCTGAGGGAACCGTGTTGT
pId1 L	CCAGTTTGCCGTCTCCAT
pId1 R	GTGTCAGCGTCTGAACAAGC
pMyoD L	GGCTTTTAGGCTACCCTGGAT
pMyoD R	TGGTGAAGAAAGCAGTCGTG
